# A Model for Identifying Optimal Patients for Primary Tumor Resection in Patients With Metastatic Bladder Cancer

**DOI:** 10.3389/fonc.2021.809664

**Published:** 2022-01-19

**Authors:** Jintao Hu, Zhenming Zheng, Junjiong Zheng, Weibin Xie, Huabin Su, Jingtian Yang, Zixin Xu, Zefeng Shen, Hao Yu, Xinxiang Fan, Jianqiu Kong, Jinli Han

**Affiliations:** ^1^ Department of Urology, Sun Yat-sen Memorial Hospital, Sun Yat-sen University, Guangzhou, China; ^2^ Guangdong Provincial Clinical Research Center for Urological Diseases, Sun Yat-sen Memorial Hospital, Sun Yat-sen University, Guangzhou, China

**Keywords:** metastatic bladder cancer, nomogram, SEER database, primary tumor resection, surgery

## Abstract

**Background:**

A survival benefit was observed in metastatic bladder cancer patients who underwent primary tumor resection, but it was still confusing which patients are suitable for the surgery. For this purpose, we developed a model to screen stage M1 patients who would benefit from primary tumor resection.

**Methods:**

Patients with metastatic bladder cancer were screened from the Surveillance, Epidemiology, and End Results database (2004–2016) and then were divided into surgery (partial or complete cystectomy) group and non-surgery group. To balance the characteristics between them, a 1:1 propensity score matching analysis was applied. A hypothesis was proposed that the received primary tumor resection group has a more optimistic prognosis than the other group. The multivariable Cox model was used to explore the independent factors of survival time in two groups (beneficial and non-beneficial groups). Logistic regression was used to build a nomogram based on the significant predictive factors. Finally, a variety of methods are used to evaluate our model.

**Results:**

A total of 7,965 patients with metastatic bladder cancer were included. And 3,314 patients met filtering standards, of which 545 (16.4%) received partial or complete cystectomy. Plots of the Kaplan–Meier and subgroup analyses confirmed our hypothesis. After propensity score matching analysis, a survival benefit was still observed that the surgery group has a longer median overall survival time (11.0 vs. 6.0 months, *p* < 0.001). Among the surgery cohort, 303 (65.8%) patients lived longer than 6 months (beneficial group). Differentiated characteristics included age, gender, TNM stage, histologic type, differentiation grade, and therapy, which were integrated as predictors to build a nomogram. The nomogram showed good discrimination in both training and validation cohorts (area under the receiver operating characteristic curve (AUC): 0.806 and 0.742, respectively), and the calibration curves demonstrated good consistency. Decision curve analysis showed that the nomogram was clinically useful. Compared with TNM staging, our model shows a better predictive value in identifying optimal patients for primary tumor resection.

**Conclusions:**

A practical predictive model was created and verified, which might be used to identify the optimal candidates for the partial or complete cystectomy group of the primary tumor among metastatic bladder cancer.

## Introduction

Bladder cancer is the 2nd most commonly diagnosed urologic neoplasm worldwide, with approximately 573,000 new cases and new 213,000 deaths in 2020 (1). And 10%–15% of patients have already metastatic lymph nodes, lungs, liver, and bone, etc., at diagnosis (2, 3). Metastatic bladder cancer (mBC) has an unfavorable prognosis, as the 5-year survival rate is only 10% (4). The current guidelines recommend that combined chemotherapy is the first-line and second-line treatment for mBC, but the median overall survival (OS) is only 15 months (5, 6). Therefore, it is very important to establish a pretreatment prediction model to accurately predict the prognosis of mBC patients.

In fact, nearly half of these patients are not suitable for chemotherapy (3). Primary tumor resection (PTR) is also one of the methods to treat malignancies and has a potentially better prognosis, but it is mostly palliative in nature (7). The purpose of PTR was to relieve symptoms and control the disease to some extent. Indeed, a survival benefit for patients with metastatic tumors after surgery on the primary site has been observed, such as metastatic esophageal cancer and metastatic non-small cell lung cancer (8–10). This may be partly because surgery reduces complications from primary tumors and benefits from multimodal therapy. Such a controversial issue also exists in the mBC field. Due to limited data and research, there is a lack of understanding of the role of surgery for patients with mBC, especially for PTR. Previous studies have found that some patients with mBC can achieve long-term cancer control with surgery (7). There is a study that suggests that retroperitoneal lymph node dissection for partial cystectomy patients with mBC has a potential therapeutic effect (11). And some studies have found that PTR combined with chemotherapy may have a better prognosis (12, 13). So far, there are still no prediction tools to identify which patients will benefit from PTR.

Hence, to address the clinical needs, we used a public database to develop and validate a novel predictive model to identify patients with mBC who could benefit from PTR.

## Methods

### Patient Selection

The Surveillance, Epidemiology, and End Results (SEER) database is a public database that contains patient baseline data, tumor characteristics, treatment, and prognosis, covering approximately 28% of the US population (14). Our study was strictly in compliance with the Declaration of Helsinki and based on the SEER database, which allowed the extraction of data (SEER-Stat username:10850-Nov2020).

Patients diagnosed with bladder cancer (tumor location coded as C67.0-C67.9) were selected during a study period of 2004 to 2016 from the SEER database by the SEER*Stat software (8.3.9) according to the primary site. The study period depends on the American Joint Committee on Cancer (AJCC) tumor node metastasis stage, where available. Also, the based clinicopathological characteristics were included (age, race, sex, histology, radiotherapy, chemotherapy, grade, TNM stage, surgical method and site, and follow-up information). The inclusion criteria are as follows: 1) bladder cancer patients diagnosed with a metastatic stage and 2) with one primary tumor only. The exclusion criteria were as follows: 1) surgery accepted is not definite and 2) patients with missing or incomplete data such as TNM stage, grade stage, survival status and time, radiotherapy, or chemotherapy.

The PTR was defined as cancer-direct surgery on the primary site, including partial cystectomy, complete cystectomy, complete cystectomy with reconstruction, and pelvic exenteration. OS was calculated from the date of diagnosis to the date of death; living patients were excluded at the time of the last recording. We hypothesized that patients who underwent PTR and lived longer than the median OS of the non-PTR group would benefit from surgery.

### Propensity Score Matching Analysis

To reduce confounding bias and facilitate matching patients in the two treatment groups, propensity score matching (PSM) was performed. Variables that could potentially influence treatment outcomes were used to generate a propensity score by logistic regression, including age, race, gender, differentiation grade, histology, TNM stage, radiotherapy, chemotherapy, and surgery to a distant site. Patients in the two groups (surgery and non-surgery) were 1:1 matched using the nearest propensity score on the logit scale with a caliper of 0.05. Subgroup analysis forest plot and standard difference were used to compare the baseline characteristics between matched groups.

### Prediction Model Construction and Validation

Based on the above assumption, participants in the PTR group were divided into two groups: a PTR-beneficial group (OS > the median OS of the non-PTR group) and a PTR-non-beneficial group (median OS ≤ the median OS of the non-PTR group). It makes clinical sense to identify the PTR beneficial to patients. Participants in the PTR group were used for analysis and split randomly into the training set and validation set at 1 to 1.

Then, a multivariable logistic regression model was developed to predict PTR-beneficial patients. A nomogram was developed based on multivariate analysis on the training set to provide a quantitative tool to predict which mBC patients will benefit from PTR. The scores for each clinical variable were calculated and summed. Thus, the total score of the patients was obtained individually. The total score corresponds to the probability that the mBC patient will benefit from the PTR. And mBC patients with a probability greater than 50% are candidates for primary tumor surgical benefit.

The prediction performance of the nomogram was evaluated by the area under the receiver operating characteristic curve (AUC) on both the training and validation sets. A calibration plot was formulated to assess the calibration of the nomogram with the Hosmer–Lemeshow goodness-of-fit test (*p* > 0.05 indicated insignificant deviance from the theoretical perfect calibration).

Decision curve analysis (DCA) is an essential statistical method to evaluate whether a model has utility in supporting clinical decisions (15). The DCA estimates the clinical usefulness of the nomogram by plotting net benefit (NB) at a range of clinically reasonable risk thresholds.

TNM staging is a commonly used predictive tool in clinical practice. In order to clarify the advantages of our model in identifying optimal mBC patients for PTR, a comparison was also made.

### Statistical Analysis

Continuous variables and categorical variables were analyzed by *t*-test and chi-square test, respectively. PSM was performed using the SPSS 26.0 (IBM Corp., Armonk, NY, USA) (16). All other statistical computations were conducted using the R software, version 4.0.2 (The R Foundation for Statistical Computing, Vienna, Austria; http://www.R-project.org). Standardized mean differences were calculated by the “MatchIt” and “rgenoud” packages. The nomogram and calibration plots were produced using the “rms,” “foreign,” and “survival” packages. The DCA was performed using the function “stdca.R.” All statistical tests were two-sided, and *p* < 0.05 was considered statistically significant.

## Results

### Patients

We extracted 7,965 mBC patients of the 3,314 patients who met the inclusion criteria. There were 545 (16.4%) mBC patients who received surgery on the primary tumor (PTR group), and 290 (69.7%) patients benefited from surgery.

**Figure 1:**
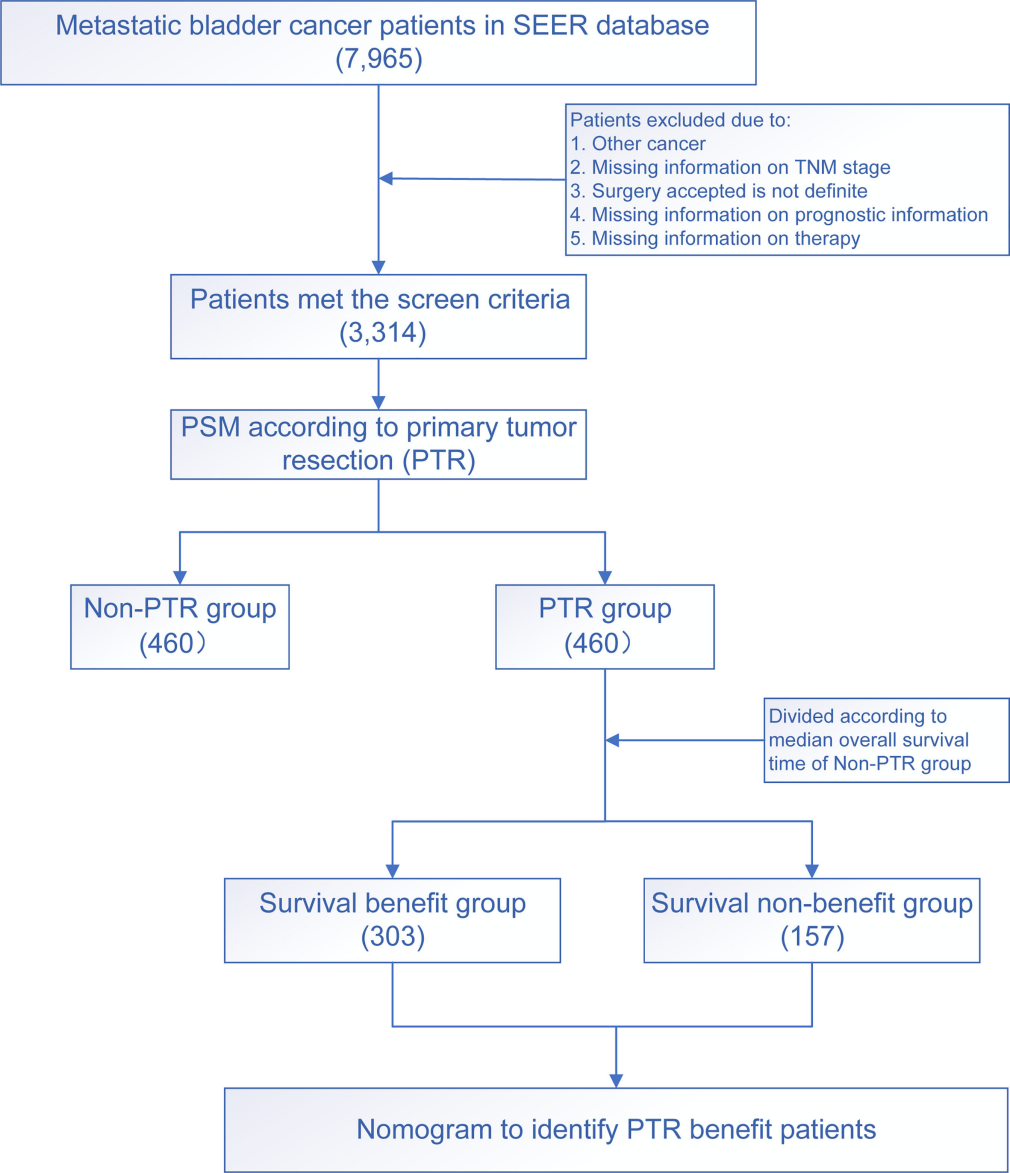
The flowchart of the study.

After 1:1 PSM, a total of 920 samples of mBC patients treated with or without primary site surgery were enrolled in the following analysis. The flowchart is shown in **Figure 1**.

**Figure 2:**
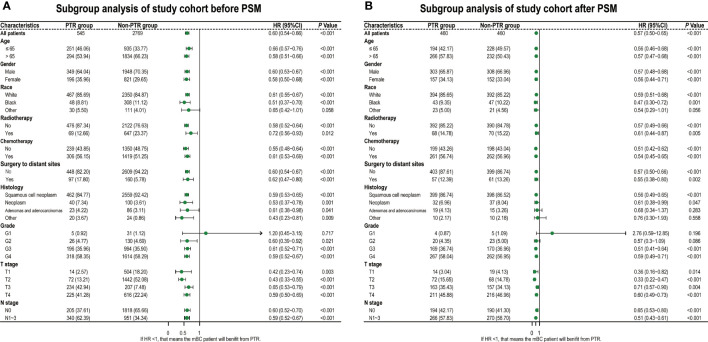
Hazard ratios of overall survival for PTR group and non-PTR groups. Diamonds represent effect size, calculated separately in different subgroups, and error bars indicate 95% CIs. **(A)** Before PSM. **(B)** After PSM. PTR, primary tumor resection; PSM, propensity score matching.

All baseline characteristics were well balanced after PSM (all *p* > 0.05), including age, gender, race, histology, differentiation, TNM stage, radiotherapy, chemotherapy, and surgery to distant sites, as shown in **Table 1**. And the comparison of the baseline characteristics between matched groups is visually displayed in the subgroup analysis forest plot and standard difference (**Figure 2** and **Table 1**).

**Table 1:** Baseline characteristics of the study population.

Variable	Before PSM		SMD	After PSM		SMD
	PTR group	Non-PTR group		PTR group	Non-PTR group	
	n = 545 (%)	n = 2,769 (%)		n = 460 (%)	n = 460 (%)	
Age, mean/SD, years	65.5/12.28	70.165/11.79	0.388	66.272/12.25	65.228/12.29	0.085
Gender			0.135			0.023
Male	349 (64.04)	1,948 (70.35)		303 (65.87)	308 (66.96)	
Female	196 (35.96)	821 (29.65)		157 (34.13)	152 (33.04)	
Race			0.101			0.035
White	467 (85.69)	2,350 (84.87)		394 (85.65)	392 (85.22)	
Black	48 (8.81)	308 (11.12)		43 (9.35)	47 (10.22)	
Other	30 (5.50)	111 (4.01)		23 (5.00)	21 (4.56)	
Radiotherapy			0.281			0.012
No	476 (87.34)	2,122 (76.63)		392 (85.22)	390 (84.78)	
Yes	69 (12.66)	647 (23.37)		68 (14.78)	70 (15.22)	
Chemotherapy			0.098			0.004
No	239 (43.85)	1,350 (48.75)		199 (43.26)	198 (43.04)	
Yes	306 (56.15)	1,419 (51.25)		261 (56.74)	262 (56.96)	
Surgery to distant sites			0.379			0.026
No	448 (82.20)	2,609 (94.22)		403 (87.61)	399 (86.74)	
Yes	97 (17.80)	160 (5.78)		57 (12.39)	61 (13.26)	
Histology			0.267			0.060
Squamous cell neoplasm	462 (84.77)	2,559 (92.42)		399 (86.74)	398 (86.52)	
Neoplasm	40 (7.34)	100 (3.61)		32 (6.96)	37 (8.04)	
Adenomas and adenocarcinomas	23 (4.22)	86 (3.11)		19 (4.13)	15 (3.26)	
Other	20 (3.67)	24 (0.86)		10 (2.17)	10 (2.18)	
Grade			0.020			0.040
G1	5 (0.92)	31 (1.12)		4 (0.87)	5 (1.09)	
G2	26 (4.77)	130 (4.69)		20 (4.35)	23 (5.00)	
G3	196 (35.96)	994 (35.90)		169 (36.74)	170 (36.96)	
G4	318 (58.35)	1,614 (58.29)		267 (58.04)	262 (56.95)	
T stage			1.396			0.067
T1	14 (2.57)	504 (18.20)		14 (3.04)	9 (4.13)	
T2	72 (13.21)	1,442 (52.08)		72 (15.65)	68 (14.78)	
T3	234 (42.94)	207 (7.48)		163 (35.43)	157 (34.13)	
T4	225 (41.28)	616 (22.24)		211 (45.88)	216 (46.96)	
N stage			0.585			0.018
N0	340 (62.39)	951 (34.34)		194 (42.17)	190 (41.30)	
N1~3	205 (37.61)	1,818 (65.66)		266 (57.83)	270 (58.70)	

PSM, propensity score matching; PTR, primary tumor resection; Non-PTR, Non-primary tumor resection; SMD, standardized mean difference.

### Correlation Between Primary Tumor Resection and Survival in Metastatic Bladder Cancer

**Figure 3:**
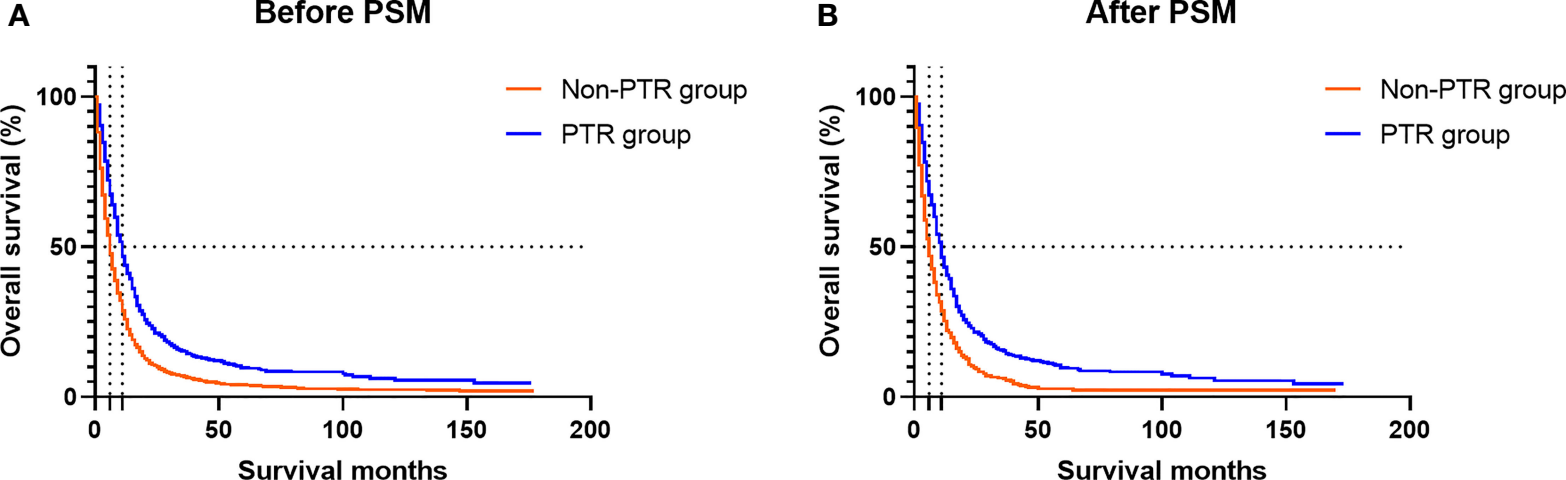
Kaplan–Meier plots show the overall survival of mBC patients according to the group. **(A)** Before PSM, the PTR group had a better prognosis than the non-PTR group (11.0 vs. 6.0 months; *p* < 0.001). **(B)** After PSM, we still observed that those who received PTR had longer median OS (11.0 vs. 6.0 months; *p* < 0.001) than those in the non-PTR group. mBC, metastatic bladder cancer; PSM, propensity score matching; PTR, primary tumor resection; OS, overall survival.

In the Kaplan–Meier analysis, significant differences in survival outcomes were observed when patients were stratified by primary site tumor surgery before and after the match. Before PSM, the PTR group had a better prognosis than the non-PTR group (11.0 vs. 6.0 months; *p* < 0.001) (**Figure 3A**). After PSM, we still observed that those who received PTR had longer median OS (11.0 vs. 6.0 months; *p* < 0.001) than those in the non-PTR group (**Figure 3B**).

As we hypothesized, based on all baseline subgroup analyses, we observed that the PTR group had a favorable prognosis with a smaller HR than the non-PTR group (**Figures 2A, B**).

### Development of the Nomogram

**Figure 4:**
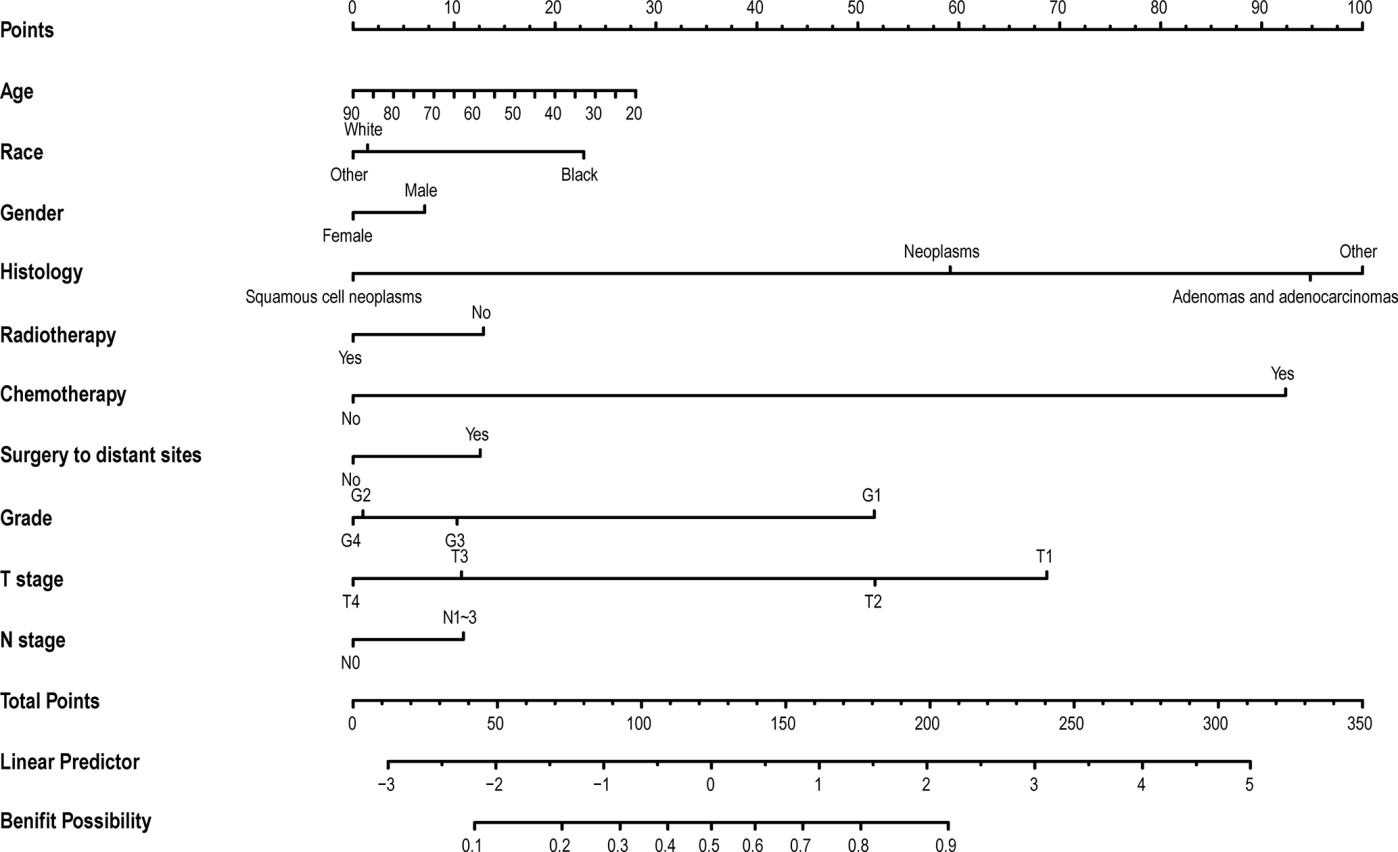
The nomogram to select optimal operable mBC patients who could gain survival benefits from PTR. The calculated score corresponds to a probability. Those whose probability value is greater than 0.5 are optimal operable mBC patients, and vice versa. mBC, metastatic bladder cancer; PTR, primary tumor resection.

In the above research, it was observed that some mBC patients could benefit from PTR. The multivariable logistic analysis revealed that age, race, gender, histology, radiotherapy, chemotherapy, surgery to distant sites, grade, T stage, and N stage were independent predictors of the prognosis of mBC patients who were received PTR (**Figure 4**).

### Validation of the Nomogram and Performance Assessment

**Figure 5:**
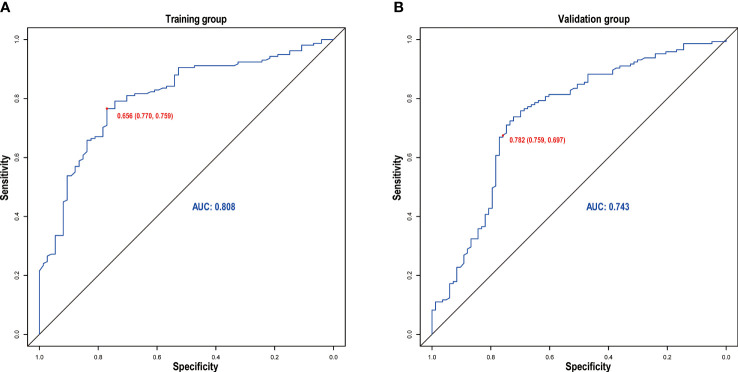
Receiver operating characteristic curve of the nomogram in the training **(A)** and validation **(B)** groups.

**Figure 6:**
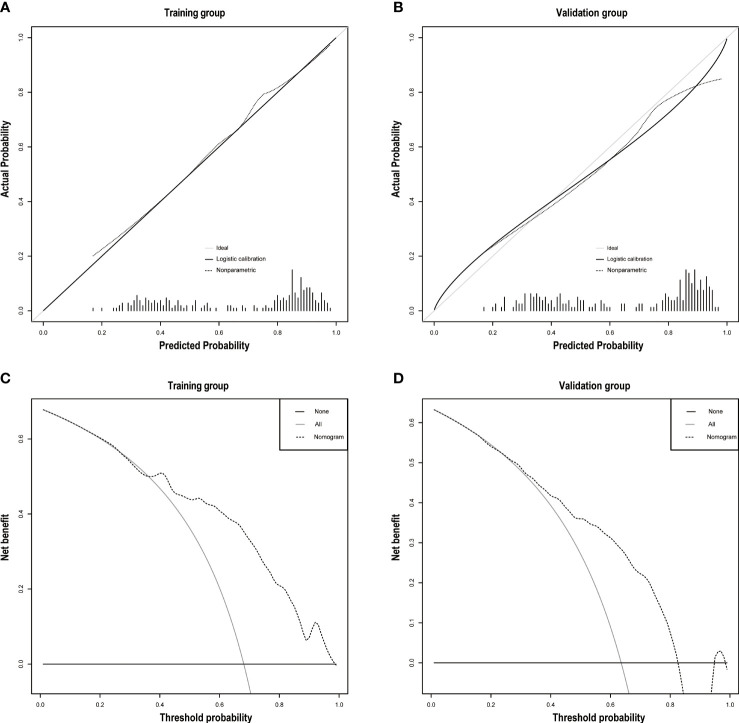
**(A)** The calibration plots of the training group. **(B)** The calibration plots of the validation group. Decision curve analyses depict the clinical net benefit in the different cohorts. On decision curve analyses, the nomogram showed superior net benefit in study cohorts across a range of threshold probabilities. **(C)** Training group. **(D)** Validation group.

The discrimination ability was assessed by the AUC index in the training set (AUC = 0.808) and the validation set (AUC = 0.743) (**Figures 5A, B**). It revealed that the nomogram had good favorable discrimination ability in the training and validation sets. Good consistency between actual observation and prediction by nomogram had been verified by calibration curves (**Figures 6A, B**). The favorable clinical practical value of the nomogram was confirmed by DCA curves in the two sets (**Figures 6C, D**).

Also, based on TNM staging, a model was established and validated (**Supplementary Figure S1**). After comparison, it can be concluded that our model has a better predictive value in identifying optimal mBC patients for PTR.

## Discussion

In this study, we observed that the mBC patient undergoing PTR had a better prognosis than the other. To identify this group of patients, we tried to build a nomogram. The nomogram incorporating age, race, sex, histology, radiotherapy, chemotherapy, surgery to distant sites, grade, T stage, and N stage had a favorable potential clinical applicability. As we know, this is the first study to develop and validate a novel nomogram to identify mBC patients who could gain survival benefits from PTR.

For mBC patients, existing guidelines recommend combination chemotherapy rather than resection of the primary site. The conventional wisdom is that PTR is mostly palliative in nature. But the value of PTR in patients with metastatic cancer has been found in several systemic tumors and likewise in the urinary system. The potential beneficial effects of PTR treatment for patients with metastatic prostate cancer mainly depend on tumor characteristics (17). In bladder cancer, surgical treatment is feasible and well controlled over time, but further evidence is needed (7). In the metastatic tumor stage, surgery could be a multimodality approach to improving prognosis (7, 18–20). In our study, which is consistent with the other studies, we observed the presence of a potential benefit from the excision of the primary tumor. To some extent, PTR could reverse immune suppression even in patients with metastasis (21). There is also literature suggesting that surgery reduces complications (8). These may be part of the reason for the better prognosis in the PTR group.

In our stable visualized nomogram, histology, differentiation, chemotherapy, T stage, and age were primary predictive variables that could estimate whether an mBC patient could benefit from the removal of the primary tumor. Our research is in line with the recent study that histology type is an independent prognostic factor for OS (22). According to our study, patients with non-urothelial carcinoma have a greater chance of benefiting from PTR. T stage, differentiation, and age are negatively correlated with prognosis. This relationship is common to non-muscle invasive bladder cancer. This fact probably indicated that the self-condition and tumor load of the mBC patients determine the outcome, which might be attributed to tumor symptoms and surgical complications.

We can still observe two therapeutic factors that cannot be ignored: chemotherapy and mastectomy. Surgery of primary tumor combined with chemotherapy may have a favorable prognosis. According to the guidelines, the first- and second-line treatment for tumors is chemotherapy (23). For mBC undergoing chemotherapy, PTR still can prolong survival time. Similar to this finding, a study confirmed that both surgery and chemotherapy are independent prognostic risk factors (22). The PTR combined with multimodality therapy could prolong survival time (24). It could be a new treatment model for surgeons but need further prospective evidence. Furthermore, our study provides a reference plan as to whether the lesions at the metastatic site need surgical resection. Surgery to distant sites may have a survival benefit. This view does cohere with David Pfister’s report that mastectomy could improve the prognosis (25).

The interesting finding is that TNM staging, although a widely used tool in clinical practice, is not perfect for all situations. Based on TNM staging, we established a diagnostic model combined with other variables. By comparison, the prediction ability of our nomogram is much better in identifying optimal mBC patients for PTR.

Since substantial heterogeneity exists, such as histology, grade and stage, and treatment, there were also differences in prognosis among mBC patients. Individualized analysis of patient outcomes and treatment modalities combined with PTR could improve the outcome of some mBC patients. So our study established and validated the first population-based nomogram to identify optimal mBC patients who would benefit from PTR. We hope to be able to further supplement the guidelines and suggest a new treatment option for optimal mBC patients. We believe that using this prediction tool in the clinic with no additional financial burden can provide a guide for surgeons in their decision making and appropriate therapy for selected patients.

Admittedly, our study has the following limitations. First, the SEER database lacks some important data, such as smoking, comorbidities, chemotherapy drugs, target therapies, immunotherapy, and location and number of metastases. Second, this study is a retrospective study requiring further external multicenter prospective validation. At present, due to the limitations of research and data, using this database is still an ideal method for our research.

## Conclusion

Our study provides a validated nomogram to assist the surgeons to select optimal operable mBC patients who could gain survival benefits from PTR. We believe our model may provide a guide for surgeons in their decision making and appropriate therapy for selected patients. And further prospective trials are required.

## Data Availability Statement

The raw data supporting the conclusions of this article will be made available by the authors, without undue reservation.

## Author Contributions

JTH, JLH, and JK conceived and designed the study, participated in the collection of data and data analysis, and drafted the manuscript. ZZ, JZ, WX, HS, JY, ZX, ZS, and HY assisted in the design of this research and project development. XF analyzed the data and reviewed the article. All authors read and approved the final manuscript.

## Funding

This study was supported by the Science and Technology Planning Project of Guangdong Province (Grant No. 2020A1515111119) and Guangdong Provincial Clinical Research Center for Urological Diseases (Grant No. 2020B1111170006).

## Author Disclaimer

The funders played no role in the design of the study, collection, analysis, and interpretation of data or in writing the manuscript.

## Conflict of Interest

The authors declare that the research was conducted in the absence of any commercial or financial relationships that could be construed as a potential conflict of interest.

## Publisher’s Note

All claims expressed in this article are solely those of the authors and do not necessarily represent those of their affiliated organizations, or those of the publisher, the editors and the reviewers. Any product that may be evaluated in this article, or claim that may be made by its manufacturer, is not guaranteed or endorsed by the publisher.

## Supplementary Material

Supplementary Figures S1
**(A)** A model was developed based on TNM staging to identify optimal mBC patients for PTR. **(B)** The receiver operating characteristic curve. **(C)** The calibration plots. **(D)** Decision curve analyses.
